# AmyloWiki: an integrated database for *Bacillus velezensis* FZB42, the model strain for plant growth-promoting *Bacilli*

**DOI:** 10.1093/database/baz071

**Published:** 2019-06-19

**Authors:** Ben Fan, Cong Wang, Xiaolei Ding, Bingyao Zhu, Xiaofeng Song, Rainer Borriss

**Affiliations:** 1 Co-Innovation Center for Sustainable Forestry in Southern China, College of Forestry, Nanjing Forestry University, Nanjing 210037, China; 2 Department of Biomedical Engineering, Nanjing University of Aeronautics and Astronautics, Nanjing 210016, China; 3 Department of General Microbiology, Institute of Microbiology and Genetics, Georg-August University Göttingen, Grisebachstr. 8, D-37077 Göttingen, Germany; 4 Institut für Biologie, Humboldt Universität Berlin, 10115 Berlin, Germany, and Nord Reet UG, Marienstr. 27a, 17489 Greifswald, Germany

## Abstract

Since its isolation 20 years ago, many studies have been devoted to *Bacillus velezensis* FZB42 (former name *Bacillus amyloliquefaciens* subsp. *plantarum* FZB42), which has been gradually accepted as a model organism for Gram-positive rhizobacteria. FZB42 is different from another widely studied bacterial strain, *Bacillus subtilis* 168, in its many features that are closely associated with plants. FZB42 represents a large group of *Bacillus* isolates that are beneficial to plants and of great importance in agriculture. In this work a database for FZB42 named ‘AmyloWiki’ is built to integrate all information of FZB42 available to date. The information includes the genomic, transcriptomic, proteomic, post-translational data as well as FZB42 unique genes, protein regulators, mutant availability, publications and etc. The website is built up with PHP and MySQL with a function of keyword searching, browsing, data-downloading and other functions.

## Introduction

*Bacillus* strain FZB42, isolated from beet rhizosphere, is a soil rhizobacterium with potent biocontrol and plant growth-promoting activities. A novel subspecies with FZB42 as the type strain, *Bacillus amyloliquefaciens* subsp. *plantarum*, was proposed within *Bacillus subtilis* complex in 2011 (1). This subspecies differs from other species/subspecies in the *B. subtilis* complex in that its members are able to efficiently colonize plant roots and contribute to plant growth (1–3). Representatives of this subspecies include a large number of commercialized strains that are developed as biofertilizers or biocontrol agents for crop or vegetation production (3–5). Obviously this subspecies represents a group of plant-associated bacteria with immense economic value in sustainable agricultural industry.

As the type strain, FZB42 plays a pivotal role in elucidation of features of *B. amyloliquefaciens plantarum*. There are more than 140 articles relating to FZB42 that have been recorded up to date (http://amylowiki.top/reference.php). Its genome was published in 2007 as the first representative of Gram-positive plant growth-promoting rhizobacteria (6). FZB42 dedicates >10% of its genome resource to encoding the genes for at least 13 antimicrobial compounds, which endow FB42 with the capability against a wide range of phytopathogens (6–8). A half of the antibiotics are non-ribosomally synthesized by a huge gene cluster that is often longer than 30 kb (7), a typical feature of *B. amyloliquefaciens plantarum* (1, 3). Extensive studies on the antibiotics produced by FZB42 have been performed in regards of their antagonistic activities, genetic basis, regulatory mechanisms and biosynthesis pathways (8–17). High throughput investigations were conducted characterizing FZB42 in different aspects: the regulated transcripts in response to plant root exudates were revealed by microarray (18, 19), non-coding regulatory RNAs and genome-wide transcriptional start sites (TSSs) were charted by dRNA-seq (20), secretome proteins and post-translational protein modifications were identified by mass technology (21, 22) and ecological impact on microbial community was profiled by metageonomic profiling (23). In addition, a dozen of studies were performed with FZB42 focusing on biofilm formation and root colonization (2, 24, 25). In general, previous work on FZB42 has accumulated a large volume of data that are critical to FZB42/*B. amyloliquefaciens plantarum* researches in future.

According to phylogenetic analysis Dunlap *et al*. (26) proposed in 2015 that *B. amyloliquefaciens plantarum* is a later heterotypic synonym of *Bacillus methylotrophicus*, which was, however, very soon (2016) corrected as a later heterotypic synonyms of *Bacillus velezensis* (27). In 2017 we also proposed to establish an ‘operational group *B. amyloliquefaciens*’ consisting of soilborne *B. amyloliquefaciens* and plant-associated *B. siamensis* and *B. velezensis* (3). The successive taxonomic changes in considering the phylogenetic clade represented by FZB42 reflect a distinct relationship of many strains within the *B. subtilis* complex. The differences between FZB42 and the model organism for Gram-positive bacteria in general, *B. subtilis* 168, forced us to build an independent platform for FZB42, presenting the data that are not offered by the several popular database committed to *B. subtilis* 168 (28–30). This effort is described in this work. We hope that the database for FZB42, AmyloWiki, may benefit many researchers who are studying on the *Bacillus* isolates with an obvious plant-associated lifestyle.

## Materials and methods

### Data sources and processing

The genomic data of FZB42 were derived from NCBI (accession No. NC_009725.1) (6). The transcriptomic data (TSSs, small RNAs, revised gene annotation) were obtained from the previous publication (20). The post-translational modifications (acetylation, malonylation) were obtained from the publication (22, 31, 32). The genes involved in microbe-plant interactions were summarized from the related publications (6, 18). The information of many common genes with *B. subtilis* 168 was obtained from the database SubtiWiki (28). In total, we collected a huge volume of different types of information, such as 3941 FZB42 genes, 4539 TSSs, 1773 protein regulators, 595 genes involved in plant–bacteria interactions and 97 FZB42-derived mutant strains. What’s more, 147 publications on FBZ42 research are provided. The above data were categorized in Excel and imported into the AmyloWiki.

### Database construction methods

**Figure 1:**
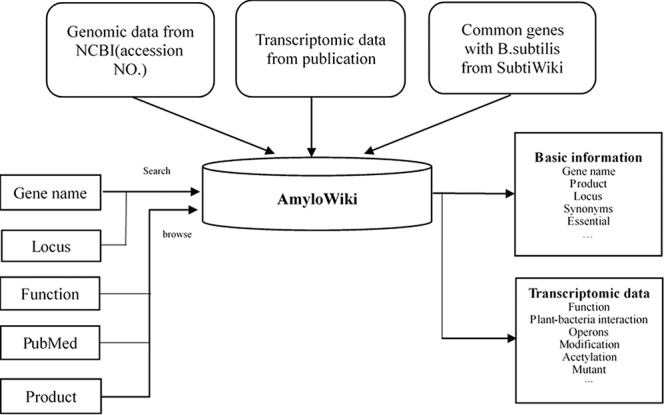
Data source and structure of AmyloWiki.

AmyloWiki was constructed and configured upon typical XAMPP (X-Windows, Linux or Mac OS + Apache + MySQL + PHP + Perl) environment. The webserver was built up with Apache 2.4.23. We collected the genomic data, the transcriptomic data, the post-translational modification data and the microbe–plant interactions information from relating publications and categorized them in Excel format. Then the file was translated into CSV format (comma-separated value file format) and imported into phpMyAdmin 4.5.1 (local database construct platform) using relative SQL command. All data sets were processed and stored in MySQL 5.0.11. Database management system and interface was achieved by PHP language (version 5.6.28). All the webpages were created with HTML5, CSS3 and JavaScript techniques. The ‘Genome Context’ section was created by *svg* label with JavaScript controlling its dynamic change. PHP and MySQL were used to process the dataset, such as retrieving a gene by keywords, browsing the dataset by specific attributes and sorting the dataset by their counts. After the database was constructed and carefully checked, the data in SQL format were imported into Aliyun MySQL server. Then the webpage file was uploaded to the server. In this way AmyloWiki is available for users to view. The data source and structure of AmyloWiki are as shown in Figure 1.

## Results

As a comprehensive and user-friendly database, AmyloWiki offers the following functions such as searching, advanced retrieval, feedback, data submission and resource downloading. AmyloWiki integrates the latest information for FZB42 including all gene context, expression and regulation information, recently identified sRNA genes, post-translational modification sites and biological materials.

### Database interface

The interfaces of AmyloWiki allow users to easily view, browse, search, download, feedback or submit data of their interest. There are seven menus at the top of the homepage: ‘Home’, ‘Categories’, ‘Regulon list’, ‘Genes not in 168’, ‘Interaction with Plants’, ‘Reference’ and ‘Resources’. There are three menus in the bottom of homepage, including ‘Contact’, ‘Feedback’ and ‘Data submission’. In the middle of the homepage, there is a text-searching box that offers ‘fuzzy’ searching function and returns all results of entries matching a keyword. Users are allowed to input gene name, gene locus or PubMed ID of related publication. There is a ‘drop-down menu’ offering the functions, such as ‘browse by functional category’, ‘browse by product’, ‘browse by PubMed ID’, ‘browse by essential gene’ and ‘browse by Sigma factors’. Below the ‘drop-down menu’, there is a brief introduction for AmyloWiki and several quick links.

### Search function

There is a text-searching box in the home page and in right sides of other webpages, where users can enter query words, such as gene name, gene locus or PubMed ID; then the items matching the query string will be listed in the result page. The users can view the detailed information about their interested gene by clicking the corresponding gene name on the left column. If there is only one result matching the key word, it will directly jump to the detailed information page. For example, if a user wants to search by gene name (e.g. *acnF*), he/she can input the keyword of ‘acnF’ or ‘acn’. Then AmyloWiki will return the genes matching the keyword. If it is the latter case, all genes such as *acnF*, *acnE*, *acnD*, *acnC*, *acnA* and *acnB* will be returned. The user can choose the corresponding gene of their interest and obtain the detailed information.

**Figure 2:**
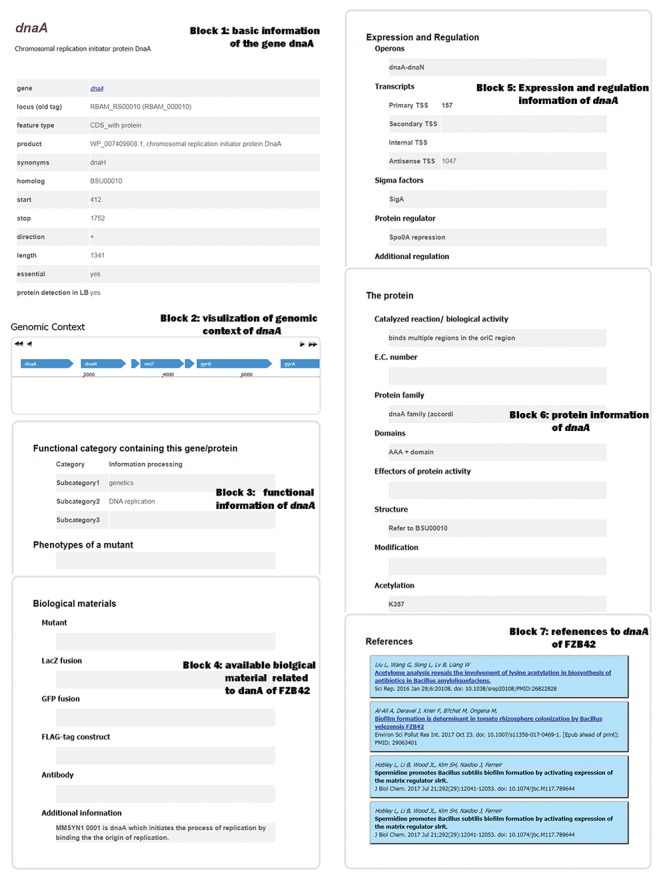
Web page for detailed information arranged in seven blocks of a representative gene (*dnaA*) of *B. velezensis* FZB42.

### Detailed information for each entry page

As specified above, users have an access to the detailed information in three ways (searching by gene name, searching by Locus or clicking the corresponding gene name in the Genomic Context). To ensure the accuracy of the database, all relevant information is kept up to date. As shown in Figure 2, each entry page is divided into seven sections. It should be noted that a blank means that information is not available. The first section provides basic information including ‘gene’, ‘locus (old tag)’, ‘feature type’, ‘product’, ‘synonyms’, ‘start’, ‘stop’, ‘direction’, ‘length’, ‘essential’ and ‘protein detection in LB’. The ‘Genomic Context’ section shows the information of location and direction of each gene in a whole genomic scale. By clicking the adjacent gene, a user can obtain its detail information, and by clicking the button above the genomic he/she can scroll up or down the genome at different speed. The third section contains the information on FZB42 genes or protein functions, phenotypes of a mutant and plant–bacteria interaction. The ‘Expression and Regulation’ section provides the information about operons, transcript, sigma factors, protein regulator and RNA regulation mode. Effectors of protein activity, protein structure and post-translational modification are displayed in ‘The Protein’ section. ‘Biological materials’ section offers relevant information about Mutant, LacZ fusion, GFP fusion, FLAG-tag construct, Antibody and Additional information. In the end, ‘Reference’ section adds the references relating to a specific gene of FZB42 at the end of the detailed information webpage.

### Data browsing

The dataset in AmyloWiki can be browsed in several options, such as ‘PubMed ID’, ‘Product’, ‘Essential Gene, ‘Functional Category’ and ‘Sigma factors’. In the home page users can select which index they want to browse the dataset. For example, in ‘Browse by Essential gene’ page, data are grouped by the essential genes, and the total number of corresponding genes is listed in the form of a table. Users can view all essential genes or nonessential genes by clicking the ‘Counts’ on the right column. Moreover, if users want to know the detailed information, they can click the gene name on the left column, it will jump to the detailed information page.

### References and data-downloading

A total of 147 publications till now about FBZ42 research are provided in the ‘Reference’ menu at the top of each page. They are sorted by the year of publication from 2018 to 1998. Users are allowed to view the literature via the links. It should be noted that in the detailed information page of each gene, related literature are also provided at the bottom.

In order to facilitate the users, all datasets of AmyloWiki can be downloaded from the ‘Resource’ page. Users can also choose the attributes of their interest and download them in an Excel-compatible format.

### Data submission and feedback

It is necessary to update AmyloWiki frequently in order to keep the database comprehensive. Thus we designed a specific page (Data submission) for users to submit their latest data to AmyloWiki. Three items (‘Gene name’, ‘Product’ and ‘Locus’) are required. Users are encouraged to input their detailed information and include their E-mail address. After checking carefully, we will add the data into the database. The ‘Feedback’ page is designed for collecting suggestions. Any ideas to improve the AmyloWiki and also to correct mistakes are highly appreciated.

## Discussion and Conclusions

In this work, an integrated database, AmyloWiki, is designed to provide comprehensive scientific data of *B. velezensis* FZB42 and to benefit all researchers across the world studying on plant beneficial *Bacillus*. AmyloWiki has collected genomic, transcriptomic, proteomic, post-transcriptional and post-translational data of FZB42 from all previous work. For genomic data, for instance, we deposited 3941 gene records, including 570 genes that are involved in bacterial interaction with plants and 432 genes that are not present in the general type strain *B. subtilis* 168. In addition, 92 records on gene mutation strains described in scattered literatures were integrated.

Although several elegant databases, such as ‘SubtiWiki’, ‘BsubCyc’, ‘SutiList’, are being widely used by researchers working on *Bacillus* (28–30), they are powerless in many cases to those working on *B. velezensis*. The latter group of researchers is in such a many number, due to the aforementioned plant-beneficial effect and thus enormous economic value of *B. velezensis*, that it is necessary to set up a platform specific to *B. velezensis* for them. As one of the most studied representative of *B. velezensis*, the biology of FZB42 has been elucidated by a large volume of data. FZB42 has also become an experimental material disseminated in tens of research groups all over the world (4). Therefore building the database with FZB42 data is urgent and highly important. Taken together, these considerations inspired us to start this work. We expect that AmyloWiki can facilitate the work of all related researchers by providing them most recent data on FZB42 and convenient interfaces for accessing these data.

Although most data hitherto on FZB42 have been collected in ‘AmyloWiki’ and functionally accessible to users, we do think that more improvements should be made in the future. These can include, for example, addition of drag and zoom in/out function to the ‘Genomic Context’ region. Most functional area will be further beautified and, most importantly, new data on FZB42 will be continuously added to the database. Improvement will also be performed on the basis of feedback of our users. We hope that AmyloWiki would finally become a powerful and popular platform for most researchers working in the field of plant growth-promoting *Bacilli*, especially *B. velezensis*.

## Notes

**Database URL**: http://amylowiki.top/
